# On Erosion

**Published:** 1884-01

**Authors:** Alfred A. Coleman

**Affiliations:** London


### On Erosion.

MR. ALFRED A. COLEMAN, LONDON.

Read before the International Medical Congress.

I too well remember having said on a public occasion "that papers written to order rarely contained anything either original or interesting," but, at the time, I little thought how soon I should illustrate my own statements. From those friends, whose persuasions led me into the scrape, I have a right to look for some indulgence, but in regard to the majority of my hearers I can only throw myself upon theirs.

Erosion or decay by denudation has had, to different individuals, different meanings, it is therefore desirable at the outset that I should at least define the subject on which I propose to treat. Thus some, Bourdet for instance, consider the former to comprehend what we, in this country at least, comprehend under honeycombed teeth, whilst Wedl describes it under the title "caries of cementum." "These carious excavations," he says, "usually have sharp edges, are pateliform, and occasionally occur without any affections of other portions of the tooth." He also evidently classes the honeycombed teeth under the head of erosion, as he states in a note with respect to these, "These malformations are considered, without reason by many practitioners, to be caused by hot drinks, sweet and sour articles of food, use of acid preparations for the teeth or other medicaments . . . . but Bourdet, who treated such deformities under the name of erosion, considered them due to rachitis, scorbutus, low forms of fever, measles, &c. . . . The surgeon Tenon exhibited to Bourdet a series of jaws, by which it could be demonstrated that the erosion took place while the tooth was still within its capsule. Again, Dr. Murinie, in an excellent paper, read before the Odontological Society, June 6th, 1870, entitled, "On some Abnormal and Diseased Dental Conditions in Animals," thus classifies the various conditions under which the teeth incur a loss of substance:

"Human teeth," he observes, "may suffer diminution in volume by four separate processes, which are respectively as follows:—

"(*a*) By true absorption, equivalent to the normal healthy process which is witnessed in the passage from the deciduous to the permanent dentition.

"(*b*) By erosion, or interstitial change of a chemico-vital character, but differing from true absorption in being the product of abnormal changes.

"(*c*) By denudation, or abrasion, simply slow mechanical rubbing away, either from contact with opposed teeth, or friction of foreign substances.

"(*d*) By force or piecemeal chopping away of portions, variable in size according to circumstances."

In this country, both terms have usually been restricted to one condition, as we shall see in the following short historical notices of it.

Hunter, who appears to have been the first to describe it, and who designated it "The Decay of the Teeth by Denudation," says, "There is another decay of the teeth much less common than that already described, which has a very singular appearance. It is a wasting of the substance of the tooth very different from the former. In all the instances I have seen, it has begun on the external surface of the tooth, pretty close to the arch of the gum. The first appearance is a want of enamel, whereby the bony part is left exposed, but neither the enamel nor the bony part alters in consistence as in the above described decay; and hence it may be called a denuding process. The bony substance of the teeth also gives way, and the whole wasted service has exactly the appearance as if the tooth had been filed and polished.

At these places, the bony parts being exposed, become brown." "I have seen instances where it appeared as if the outer surface of the bony part, which is in contact with the inner surface of the enamel, had first been lost, so that the attraction of cohesion between the two had been destroyed, and as if the enamel had been separated for want of support, for it terminated all at once. "In one case the two first incisors had lost the whole of the enamel on their anterior surfaces; they were hollowed from side to side, as if a round file had been applied to them longitudinally, and had the finest polish imaginable. The three grinders on each side appeared as if a round file had been used on them in a contrary direction to that on the incisors—viz., across their bodies close to the gum, so that there was a groove running across their bodies which was smooth in the highest degree. Some of the other teeth in the same jaw had begun to decay in a similar manner; also the teeth in the lower jaw were becoming diseased.

"I saw a case very lately where the four incisors of the upper jaw had lost their enamel entirely on their anterior surfaces, and there was scarcely a tooth in the mouth which had not the appearance of having had a file applied across it close to the gum.

"Those whom I have known have not been able to attribute the disease to any cause; none of them had ever done anything particular to the teeth, nor was there any appearance particular in the constitution, which could give rise to such a disease. In the first of these cases the person was about forty, in the last about twenty years of age. From its attacking certain teeth rather than others, in the same head, and a particular part of the tooth, I suspect it to be an original disease of the tooth itself, and not to depend upon accident, way of life, constitution, or any particular management of the teeth."

I have quoted Hunter at considerable length, and for the reasons that, of those who ever wrote on medical subjects, probably no one ever surpassed him in his remarkable powers of description, and because my so doing will save me from any attempt to define what I regard as covered by the designation of decay by denudation.

Fox, some thirty years later, thus writes respecting it, under the designation "Of the Removal of the Enamel by the Denuding Process." This is a disease producing a change in the teeth by which they acquire an appearance unlike that of caries, but attended with a loss of substance.

"The tooth does not, as in caries, become softer, nor, like that disease, does it originate in inflamation, but it consists in a removal of the enamel from the bone of the tooth, as if by solution and gradual abrasion.

"The first appearance is in the enamel of one or more of the incisors becoming thinner, and appearing as if a small portion had been scooped or filed out, occasioning a slight depression. This removal of the enamel continues until so much is taken away as to leave the bone exposed. As this denuding process, according to Mr. Hunter's term, advances, the tooth changes its color, gradually becoming yellower, as the bony part is more exposed. When the whole of the enamel is destroyed, part of the bone is also removed, the remainder acquires a brownish hue, is very highly polished, and will often remain in this state for a number of years.

"In some, it appears near the neck, the cavity extending across the tooth, and the disease proceeds until the tooth is nearly divided in two; in this case, whilst biting something hard, the lower part of the tooth usually breaks off, leaving the fang in the socket, and a small portion of the body of the tooth. The molars are more subject to this disease than any of the other teeth. The incisors of the upper jaw are very frequently affected by it, whilst the incisors of the lower jaw seldom become decayed."

Mr. Bell, under the title "Loss of Enamel and Wearing Down of Teeth," thus speaks of it: "Mr. Hunter appears to have been the first writer who described the remarkable, and, hitherto, unexplained affection, termed by him "Decay by Denudation.' It consists of a gradual loss of substance, generally without the slightest discoloration or any diseased appearance. In all the cases in which I have been able to observe its commencement, it appears to have begun on the surface of the enamel; in most it attacks, first, the central incisors, sometimes removing the anterior surface of the enamel, either generally or in irregular patches, in either cases making a perfect and regular horizontal groove, which very gradually increases until it has extended through the enamel to the bone." "The groove is perfectly straight and continuous through its whole extent, and is often as regular and smooth as if it had been formed by a fine round file, and afterwards polished; when the bone has been exposed by this process it sometimes becomes very slightly discolored; but remains for a long time perfectly hard and sound and seldom exhibits any appearance of gangrene. . . . The most obvious solution," *i.e.*, of the question of its cause, "would be, that it was the result of friction, and I have seen so many instances in which it has appeared to be produced, or at least increased, by the use of rough or acidulated tooth powders, that I should not hesitate to assign to it such a cause, were it not for certain cases which have exhibited this affection to a considerable extent, without any application of that kind having been ever used."

On a discussion upon this subject at the Odontological Society, May 2, 1870, in reference to a case brought forward by Mr. Steele, Mr. Harrison remarked: "That he had seen, in the course of his practice, many similar cases to that which had been brought under their notice by Mr. Steele, and had come to the conclusion, where they could not be traced to mechanical causes, that they were referable to the cause (be it what it might) which produced that effect on the teeth generally known in the profession as "Hunter's Denuding Process," which he believed was a disease—if it might be called a disease—the cause of which had not yet been discovered. This view of the subject opened, to his mind, a very interesting question for their consideration, but one which, of course, they could not go into at any length that evening, as they had before them a paper on a different subject for which the evening had been set apart. He might, however, perhaps be allowed to state a few facts which had come under his observation, with reference to this disease, or peculiar condition of the teeth so frequently met with, as the subject had turned up incidentally, which he thought would be interesting to the Society, and which he would not occupy much time in doing. It was, as they all knew, attributed by most modern authors to the use of the tooth-brush, abrading tooth-powders, and other similer mechanical causes. That they were constantly meeting with cases of ordinary abrasion of the necks and of the surfaces of the teeth produced by these causes he (Mr. Harrison) was ready to admit; but he had never been able to consider the peculiar affection described under this name by Hunter as being produced by these causes, because they were constantly meeting with cases of it in which the depressions were so deep, as compared with their breadth, and in which, moreover, they were sometimes so sharply undercut, that it had always appeared to him impossible that they could be produced by the use of an ordinary tooth-brush and any tooth-powder; and he had not been many years in practice before he became convinced of this, finding well-marked instances of this disease, or peculiar condition of the teeth, in some of the lower animals. After much consideration given to the subject, he had, at one time, come to the conclusion that it must be produced by the action of certain absorbents in the teeth themselves, not yet discovered; and he had nearly settled down into that belief (although there were almost irreconcilable difficulties in the way of it), when the following curious case caused him to suspend, if not to relinquish, this idea. A patient of his had worn for some years a complete upper and a partial set of artificial teeth, when, in consequence of having to lose some of her remaining under teeth, he made her a lower row, carved out of the hippopotamus tusk, to wear temporarily until the gums should be in a state to receive a new lower set of a more permanent character. She was so comfortable, however, with this temporary set that, instead of wearing it for a few months only, as was intended, she wore it for eighteen months or two years, when she came to him to have the permanent lower set made. On removing this temporary set from the mouth, he was at once struck with the appearance presented by its front teeth—they having a number of transverse lines of indentation running across them, from about canine to canine, exactly resembling this so-called "Hunter's Denuding Process." He drew the patient's attention to these lines, and asked her whether she had cleaned these teeth with any particular tooth-powder, or in any other particular way, which could have produced this effect. Her reply was—"That she had not, and that she had merely washed them with her tooth-brush and water with occasionally, perhaps, a little soap." He then asked her whether she had been in the habit of rubbing the front teeth more freely and with more force than the side teeth; but her reply was — "No, that habit, if anything, of rubbing the side teeth most freely, inasmuch as they had soon become the most discolored." Struck with the similiarity of these lines to what was known as "Hunter's Denuding Process" (as he had already said), he was set speculating as to the probable cause of them; and the best conclusion he could come to on the subject was, that they were in all probability produced by some peculiar action of the secretion, or of the absorbents, or of both, of the mucous membrane of the lower lip, and that, if so, "Hunter's Denuding Process" might arise from the same cause; and thus it was that this case went far, at any rate, to upset his previously existing notion that the latter disease arose from the action of undiscovered absorbents in the teeth themselves, if it did not entirely dissipate it. He begged to be understood, however, as throwing out the latter view merely as a suggestion, and not as a decidedly formed opinion; and would be glad if, in the interest of science, some enthusiastic physiologist and pathologist amongst them would take the subject up, and endeavor to work it out.

Messrs. J. and C. S. Tomes in a "System of Dental Surgery," second edition, give a good description of the appearance presented in the affection. They say: "Now and then the teeth are attacked in portions inaccessible to the tooth-brush; thus in the canine here figured, the groove was not only upon the anterior surface, but extended back on both sides of the neck of the tooth. Moreover, it was distinctly undercut. . . . Although the labial surfaces of teeth are usually attacked, the lingual surfaces may, in some few instances, be eaten away; in the two incisors here figured this has taken place, the gum having at the same time receded. The cause of these various forms of erosion has been from time to time the subject of great discussion; though some hypotheses, such for instance, as that which attributes it to disease inherent in the dentine, may at once be dismissed. It has been already shown that mechanical abrasion will not serve to account for all the cases. Absorption can not be called in to account for the removal of the tooth substance, for it often takes place at spots remote from any structure capable of developing an absorbent organ, and it seems that we must fall back upon the idea that it is an example of chemical solution. But whence the solvent comes, and why the affected surfaces are not the site of ordinary caries, are questions which remain unsolved, though it seems probable that mucus, by fermentation or affording a nidus for fermentation, may provide an acid solvent." Under the head of abrasion the same author writes, "Another form of abrasion is that which is occasioned by the action of the tooth-brush on the necks of the teeth, or upon such parts as are but indifferently protected by the enamel of the gum. The teeth become very gradually indented by highly polished transverse grooves."

The occurrence of such grooves has been by many writers supposed to be independent of a mechanical cause. But in many cases, which have come under my own notice, the grooving could be attributed to the action of the tooth-brush, or to mechanical action, in some other form. The most prominent teeth are invariably the most deeply cut, or those teeth in which the enamel is obviously defective.

Dr. Magitot thus describes the affection, and suggests its probable cause: "The cavity then presents the peculiar appearance which may be compared to a saw-cut, polished, hard, and resistant. Such are the cavities which Duval and several other authors have designated under the name of 'Caries Simulating a Wearing Away,' and of which the mode of production has not been explained. They have in effect all the appearances of true wear, but my observations upon the succession of different periods in the malady have demonstrated that these clean and polished notches are nothing else than caries of the neck of the tooth passed to the condition of spontaneous cure or dry caries."

Salter in his "Dental Pathology and Surgery" does not allude to the affection.

From the quotations I have given, from the works of chiefly English writers, it will, I think, be admitted that they do not materially differ in their descriptions of the appearances the affection presents; their differences appear when they come to assign to it a cause, and here no doubt their opinions are worked by their own special views with regard to dental caries, and with which they, more or less, associate this. In one respect, however, their differences are somewhat marked, namely, with regard to the various teeth that are most liable to it. Hunter, whilst stating that it attacks certain teeth rather than others, does not specify which; but if we take the cases he cites as typical ones, it would appear to be the incisors and molars. Fox, apparently, contradicts himself on this head; thus, he says, in one place, "The first appearance is in the enamel of one or more of the incisors;" whilst later occurs the remark, "The molars are more subject to this disease than any of the other teeth. The incisors of the upper jaw are very frequently affected by it, whilst the incisors of the lower jaw seldom become decayed." Bell says, "In most it first attacks the central incisors."

My own observations would lead me to differ somewhat from the above-mentioned authorities, and to assign to the bicuspides and cuspidati the greatest liability to the condition. That it is more common to the teeth of the upper than to those of the lower jaw is the opinion of J. Tomes, and I believe of most who have described the affection.

With the exception of Fox and J. Tomes, none of the above speak of a termination to the process other than as becoming arrested when the part affected assumes a brown color; nor do they allude to the almost invariable coincident of secondary dentine, which obliterates the pulp-cavity, and prevents exposure of the pulp. I believe it to be extremely rare for the pulp to be so exposed; when it is, it will be generally found that it has resulted from true caries having supervened upon the denudation process.

With regard to age, it is certainly an affection more common to middle life than to any other period. Hunter speaks of his two most interesting cases in individuals aged respectively twenty and forty. I should say that at about the intermediate period it is generally met with. If it occur in the milk set, it is certainly rare. I cannot say that I have never seen it, on the contrary, I believe I have ; but what I have seen may have been only that superficial form of decay so common to the first teeth--and which might perhaps be well designated erosion—which has become arrested and polished by friction. In looking over a large collection of temporary teeth, I could not find a single instance of it, although the sharply defined line at the limit of the enamel, especially on the lingual surface of a molar, might, to a careless observer, lead to such an error.

*Sex.*--Any really careful observation I may have made, with respect to the affection, dates from so short a period that, if I venture to offer an opinion upon its relative frequency in the two sexes, it must be regarded as little other than a vaguely formed opinion, and if I express such, it is that I incline to the view that it is more frequently met with in females than in males; but my impressions on this head may have arisen from the fact that we see more female than male patients.

The appearances presented by the affection have been so well described by Hunter and other writers quoted, that, as before stated, there is no necessity for my recapitulating them ; I could only wish it were as easy a matter to assign to it its true nature and cause. I have been able to make out but little that is really satisfactory to my own mind, still, as that may perhaps prove one step further in knowledge gained, I willingly, though diffidently, communicate it.

With regard to microscopic observations—and here let me state that without, for one moment, undervaluing that immense means for extending and confirming our knowledge of both physiology and pathology, yet the appearances presented by highly-magnified sections are so deceiving, that even the honest inquirer going to them with strong preconceived notions is very likely to make out with his eyes that which his heart longeth for. With regard to myself, however, I may say that what I might have expected to see that I could not make out, though it is very probable that what I may describe is not that which I actually did see.

Section 1, of which with three others I have given illustrations, together with the outlines of the teeth from which they were cut—and which must be regarded as decidedly diagrammatic rather than literally correct, as you will see when inspecting the originals under the microscope—is from an upper left central incisor kindly sent to me by Mr. Pearman, of Torquay, and is a well-marked specimen of the affection, but, as is not uncommonly the case, has suffered at an advanced stage from true caries as well. This is shown in the upper part of the diagram, and which resembles an ordinary though not characteristic specimen of caries. The tubular structure approximating upon the carious surface is rather more conspicuous than in the healthy portions, and terminated in an undefined mass of yellowish broken-down structure, mixed up probably with micrococci. In the lower portion, and which is not affected with caries, a sharp clear edge is presented externally, whilst it will be remarked that the dentinal canals are of uneven calibre and present a beaded form, the contents being apparently very unevenly distributed throughout them.

Section 2 is taken from an upper cuspidatus of the left side. In this we see the same varicose and beaded condition of the tabuli as in No. 1, but we see also, that which in the small number of sections I have been able to make is as often absent as present, that is, some appearance of the "zone of Tomes;" in no specimen have I found it so well marked as to be able to say that it was identical with it, and as the nature of this phenomenon, both as an evidence of antagonistic energy to disease, or a manifestation of disease itself, is disputed, I feel that I shall gain little in insisting or otherwise in its appearance on the decay by denudation.

Section 3 I regard as a very interesting one, being a lower left cuspidatus, for a long while the only tooth in the mouth of an elderly gentleman whom I had under observation for several years. At the back of this tooth there rested a vulcanite plate, and its continual movements wore the tooth half through, the pulp-cavity, which is not quite obliterated, being protected only by a very thin wall of secondary dentine; besides the friction of the plate, which has kept the surface as highly polished as in almost any case of decay by denudation, I think we ought to add the softening action of particles of food which lodged upon the portion of the plate in contact with the tooth—I regret the section as cut shows too much of transverse structure of the dentinal tubuli, but still I think it can be clearly seen that the dentine appears perfectly healthy, and is quite free from the characteristics of caries on the one hand, or the beaded condition of the denudation cases on the other hand.

Section 4 shows in a marked degree the beaded contents—or non-contents—of the dentinal tubuli. In some cases this is so marked as to prompt the suggestion that they may have undergone a fatty degeneration. I am well aware that such an appearance might be produced by other conditions, as the presence of air, or otherwise, in the interior of the tubuli; but, if so, it is difficult to account for its almost complete absence in the friction-produced specimen, No. 3. The section is obtained from a left lower cuspidatus, also kindly sent to me by Mr. Pearman, of Torquay, and is in more respects than one an interesting specimen; it is more or less coated with tartar upon all portions of the crown, and fang, excepting the external surface of the former and a special portion of the latter. The affected portion, at the neck, is well marked, and might serve for a typical specimen of it. But that which is of chief interest is a small, scooped out, highly polished facet, continuous with, but below, the above mentioned, and approximating upon its posterior or distal surface. I should have much liked to have seen this tooth *in situ*, but even the donor states "I have no history to give with them."

Section 5, which I have not illustrated by diagram, is from an upper right molar, most probably the first of that order. It presents the affection as a sharply defined notch at the neck, and chiefly above the posterior fang. It is this form which is so highly suggestive of the action of the tooth-brush, though found in those to whom, if this appliance be not unknown, its use is not familiar. It would, however, be interesting to ascertain whether it exists in those who do use, and in those who do not use, the tooth-brush, in the same proportion. The section possesses much the same characteristics as those before described, only in this one are to be seen faint markings of inter-globular spaces not met with in the others.

The conclusion I have arrived at, from what I must confess to be other than a deep investigation of the subject, are firstly, that the main element in the process is friction, but that the condition which permits the loss of substance thereby is favored by a change or degeneracy in the tooth itself. We will consider these conditions in the reverse of the order above given.

My impressions, previous to commencing the present investigation in regard to the affection, were that it arose from the direct action of some solvent furnished by the labial glands, or those of other parts which were immediately adjacent to the wasted spots. The application of litmus to both these spots and the opposing glands or surfaces, however, has never, in a large number of cases, afforded any greater evidence of acidity or alkalinity than is to be found in normal saliva in its varying conditions. I am well aware that solvents of tooth structure might exist having neither of those reactions; indeed my own views in respect of dental caries point in such direction. But then one would naturally expect to find more or less accumulation of foreign matter at those places, whereas the teeth there appear more free from deposition than at any other portion of their exposed surfaces. Again, is it reasonable to suppose that a substance composed of such material as a mixture of gelatinous matter and lime salts should, when attacked by a solvent, unless accompanied with considerable friction, present a very hard and highly polished surface. I cannot avoid the conclusion, but that in the majority, at least of denudation cases, some degenerative changes have taken place in the tooth structure, and especially in that of the dentine, and which has the effect of rendering them less capable of resisting friction than when in a normal condition.

It would be pure speculation on my part to attempt to suggest how this might be brought about; but if I did so speculate, I might suggest whether, by a fatty degeneration of the contents of the dentinal tubuli, and the formation of a fatty acid, the surrounding phosphates of calcium might be influenced; or, from another point of view, which embraces ideas which are more and more growing out of favor, as being less in accordance with facts elicited by modern research, it might result from a lessening of that power of resistance vaguely termed vital force, which has been supposed capable of protecting the bodies it pervades from chemical and physical influences.

A fact of no small significance in relation to this subject is the relative liability in certain teeth to be worn away, or otherwise, under almost precisely the same conditions--viz., such as food, age, habits of life, etc. Amongst a class of people who reside in a country where a large proportion of grit finds its way into their food, as in the case of the Bedouin Arabs for instance, we shall find the teeth almost invariably much worn on the masticating surfaces; but yet the differences in degree are so marked as to lead irresistibly to the conclusion that some teeth resist this attrition better than others. If we were to take a large number of individuals living in this country under precisely similar conditions, I more than predict that we should find considerable differences in the degree to which their teeth had resisted the friction of mastication. All of us must have been at times struck with the apparent wear of teeth at spots and places, where, if from friction, it could only have been that from the tongue or the larger fragments of food. Here are casts illustrating this point, kindly furnished to me by an intelligent pupil, Mr. C. E. Truman. It is clear, I think, that the portion of the surface of the molar marked could never have met an opposing tooth. But the most marked case of this kind which ever came under my notice occurred in the person of a clergyman, for whom I constructed a denture, supplying the six front teeth, and, I believe, also all the bicuspids of the upper jaw. The teeth whose places were supplied were all so perfectly level with the gum that there was no necessity, I well remember, for any employment of the file or other appliance for reducing their surfaces. The bite was certainly an edge to edge one; but the individual could not have been past forty. I much regret that I parted with the model of this interesting case. There was nothing in his history to account for this unusual wearing away of the teeth, and I think we can only conclude that his teeth, and the teeth of like condition, must be, in certain respects, different from those in other persons.

With regard to the agency by which the friction is, in all cases, furnished, I shall, in accounting for it, as you will surmise, encounter some difficulty. This, however, to my own mind, has gradually lessened as my investigations have progressed. With regard to how furnished, this, I think, may be speedily disposed of by the recognition of the act that all the movable soft structures of the mouth—but the tongue especially—are capable of reducing in dimensions the dental structures. We well know how soon a sharp edge of enamel is reduced by the movements of the tongue; the latter suffers in the contest, but finally prevails, in consequence of its powers of repair. This upper model shows how a tooth may suffer from the constant rubbing of a thumb, in the evidently gratifying process of thumb-sucking; between cause and effect in this instance not a shadow of doubt exists. An indirect point in this case is that the individual of whom this cast is the representative, and who is a child of eight years and a half old, has, as it will be observed, all her lateral incisors wanting, and hence it may also happen that the incisors she possesses may be more or less abnormally imperfect in development; also, it may occur that the degenerative changes that induced the affection may be similar changes to those which occur in the temporary teeth prior to their absorption. The teeth, the fangs of which present a horny appearance—*i. e.*, where the tubular appearance is obliterated—are, we know, peculiarly subject to absorption; and most of the teeth affected by denudation which I have examined, present more or less the horny character, some in a considerable degree.

An intelligent friend, if, indeed, I can call the individual "friend," who chiefly persuaded me to commit myself to this paper, but who, as a careful observer, is entitled to much consideration, informs me that he believes he never witnessed the process in a necrosed tooth. I am sorry I can neither oppose nor confirm Mr. Campion in this view, although I rather incline to his opinion. In some of the specimens before you the teeth were most probably dead at the time of their removal, but the process may have existed only at the time they possessed vitality. If such be the case, I think it rather tends to support the view of there being a degenerative change in the contents of the dentinal tubuli affecting the surrounding dentine, and which, ceasing with the death of the tooth, the process is arrested. A condition not much dwelt upon by writers upon the subject is the great sensitiveness presented at the denuded spots, especially in the earliest stages; when touched by the finger-nail, or hairs of the tooth-brush, they give a sharp and peculiar thrill, with a sensation like cold along the region of the spine. A tooth under such conditions cannot be devoid of vitality. Another point, also bearing on this opinion, may be mentioned, and it is the only one I will offer with regard to treatment; for though I believe the plan will invariably, if persevered in, prove successful in arresting the affection, and causing it to assume the brown color mentioned by the earlier observers as the evidence of its cessation, it is of too empirical a nature to come under the head of a rational treatment. It is the constant local application of ammonia or one of its compounds. I usually recommend sal volatile—as being of a suitable strength—to be applied on a little cotton, or brush, three or four times in the day. If, as suggested, a softened condition in the dentine exists, and if this be brought about by an acid condition of the contents of the dentinal tubuli, then a limpid alkali, and such an one as can penetrate the tubuli, would be the most likely to counteract the condition.

To return to the question of friction—and here I feel I must differ from those who point to its existence—I mean the affection as clearly described by Hunter—in places inaccessible to any agent capable of rubbing or touching the same. I believe I have never seen a case in which it was not possible for friction, either by tooth-brush, tooth-pick, tongue, lips, or saliva, to have been applied. With regard to the last-named, my friend Mr. Todd has reminded me of the familiar instances of the stone worn by the continual dropping of water, and the pebble rounded in the brook; as also of the fact that all the pebble ornaments, pen handles, etc., produced so cheaply in Germany, are polished under water. In my own mouth are some well-marked cases of the affection, but from their dried surfaces my tongue and lips can at once remove any pigment applied to them. In respect of those cases which present fine saw, cut-like markings on their approximal surfaces—

molars especially--near to the gum, they could certainly most readily have been produced by some hard-pointed instrument, continuously applied in the same direction, with the object of pressing food from the buccal to the lingual orifice of the space existing at the necks of such teeth when the gum has receded. Were such markings found amongst the teeth of the lower animals, I should then at once withdraw a tooth-pick origin to account for them; but what does exist, so far as I have been able to ascertain, of an approach to this affection amongst the lower animals, I will now endeavor to describe.

Through the kindness of Mr. C. S. Tomes, my attention was directed to the peculiar markings on the tusk of the female Indian elephants, "Specimen--2769A in the Museum of the Royal College of Surgeons." A history attached to these or similar specimens will be found in the Transactions of the Zoological Society for the year 1871, page 145.* The elephants bearing these tusks were shot in Ceylon by Mr. Sclater, and at the time he remarked the peculiar and unusual markings upon them; also a mass of egg-like bodies, arranged in regular series, of apparently some dipterous insect, somewhat resembling those of the common blowfly. On examination of the grooves at the gum no maggots were discovered. The subject has, I believe, been further investigated by Dr. Spencer Cobbold, and discussed at the Linnæan Society, but I have not been able to find any paper on the subject in its Transactions. Being unable to ascertain at the College of Surgeons how much of such tooth would be exposed above the gum, I visited the gardens of the Zoological Society, and there inspected the mouth of a female Indian elephant, which showed that all the grooved portion was situated below the gum, and the smooth portion above. The affection, therefore, as shown in one of the casts I was kindly permitted by Professor Flower to take, was, probably, at least some portion of it, below the level of the gum, and had, I should apprehend, undergone absorption, from either some altered condition of, or secretion from, that structure or the alveolo-dental membrane; * but how far this might be due to the

* Dr. Murie, *op. cit.*, remarks that it is "a case where, more truly, erosion from periosteal inflammation is indicated."

presence of the parasites, or the parasites owe their existence to it from the presence of some unhealthy secretion, is a matter upon which I will not venture to offer an opinion. I think I may, however, remark that the conditions presented by these elephants' tusks resemble but very slightly, if they do at all, the condition of decay by denudation as witnessed in man; nor, on the other hand, do they resemble ordinary dental caries. With regard, however, to the teeth of a certain class of seals, the otaria jubata (sea-lion, or fur seal,) the case is different; here we have, judging from external appearances only, a condition similar to, or approaching, that seen in the human species. With respect to this condition, Dr. Murie, in the paper before referred to, remarks: "To my mind, neither the supposition of inherent disease, nor simple friction, of a certainty accounts for the uncommon manner in which the otary's teeth have lost substance." It is possible, he admits, for the dental apparatus to be worn away by, or denuded of, the softer dentine by the unequally fitting and rubbing of the teeth against each other; but in the case of the otaria he believes this impossible; "for the abraded surfaces are not those which meet in mastication or closure of the mouth." My conclusions in this respect, however, somewhat differ from Dr. Murie's. The immensely broad condyles of the lower jaw show a great capacity for lateral movement, and such as would, in the recent state, almost permit the rotation of the lower around the upper cuspidati, and I think there can be no doubt but that much of the loss of these teeth, at least, is due to antagonistic friction. It is also a fact, as pointed out by Dr. Murie, "that seals—notably the species under consideration—swallow mouthfuls of pebbles." . . . "Now granules of sand may be taken up at the same time, and effect an erosion equivalent to an injurious dentifrice," but he considers the staining, regular circular abrasion, nature of the polish, and the mode in which particularly the great canines are reduced, oppose rather than support mechanical friction alone as the agent at work. He does not believe it can be due to absorption, nor does he see his way clear for accepting inherent disease of the tooth-substance as a cogent reason for the removal of the tissue. "By a combination of them, however, the resultant wearage is more likely to have been brought about." He suggests "an inherent constitutional tendency for the teeth to be acted on by external agency." . . . . "An altered or unhealthy condition of the glandular fluids," in which "the lips, gums, and tongue, would play a vital part." . . . . "Add to this the mechanical influence of fine particles of sand, grit, etc." He neither explains nor contradicts the assertion that certain cases of notching and erosion may be due to purely mechanical agencies; indeed, he states his belief that such instances do occur; at the same time he is none the less convinced that examples, both in men and animals (the otary to-wit) show that dental loss happens through processes other than friction *per se.*

I am, I confess, at a loss to explain this condition, as occurring in the seal, by any reference to a degeneracy in dental structure, such as we can justly assign to a being like man in the civilized condition, and in whom we should almost expect to find organs so imperfectly employed as are his teeth in a state of degeneracy. And in his case, with regard to the question of abnormal friction, surely the fact of man's being endowed with speech must be taken into account. Speak but for a short time with your attention directed to the rapidly continuous movements of the lips and tongue, and then consider what that friction under water must amount to in but one year. It is an interesting fact, that of all animals, the sounds which can be made by the seal most nearly, I believe, resemble the human voice, so that it has often been called "the talking fish." Its tongue is long and free, and although it appears to swallow its food with but little mastication, the tongue, though I have not been able to verify in my examination of the seals—which are not the furred variety—at the Zoological Society's Gardens, may be well employed in removing the scales of fish which no doubt at times adhere to the teeth with some pertinacity. It is such a tongue as can well sweep round its cuspadati, premolars, and molars, which have considerable distances between them.

When speaking of the affection as being more common to the female than to the male sex, I trust my hearers will freely acquit me of having, at the time, this suggested agency for it in mind, an agency which, if it have a true existence, must be regarded as another element in our civilization tending to bring the *genus homo* to an edentulous condition.

When I look round upon this assembly and this Congress, numbering so many distinguished individuals, and from such varied countries, a temptation arises which it is difficult to resist.

I can conceive of no grander opportunities than are those afforded by these International Medical Congresses, for the general acquiescence in, and settlement of, a universal nomenclature of the diseases which are common to the countries represented; and if I may be so bold as to make a suggestion, it is to the fact, that this International assembly, of dental physicians and surgeons, do herewith decide upon the designation which, in their opinion, may best describe the affection first noticed by Hunter and by him named "The Decay by Denudation."

